# Supplementary material for: A Sweet Potato MYB Transcription Factor IbMYB330 Enhances Tolerance to Drought and Salt Stress in Transgenic Tobacco
Source: Genes (Basel). 2024 May 26;15(6):693. doi: 10.3390/genes15060693 (PMC11202548; doi:10.3390/genes15060693)
Supplement: Supplementary file 1 [file genes-15-00693-s001.zip › FigureS2.pdf]

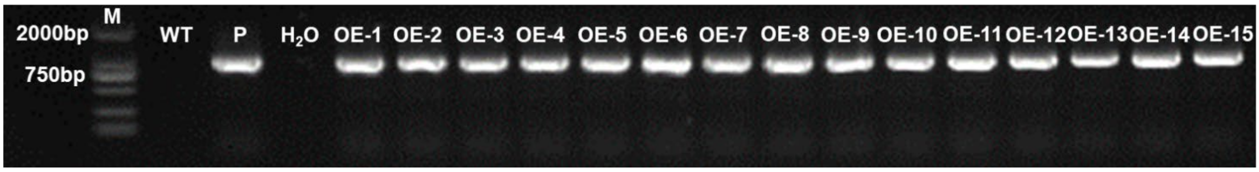

Figure S2. PCR Detection of *IbMYB330* overexpression tobacco. OE-1-15: *IbMYB308* transgenictobacco; WT: wild-typetobacco; P: overexpression plasmid of *IbMYB330*. M: DNA Maker DL2000.
