# Supplementary material for: A Sweet Potato MYB Transcription Factor IbMYB330 Enhances Tolerance to Drought and Salt Stress in Transgenic Tobacco
Source: Genes (Basel). 2024 May 26;15(6):693. doi: 10.3390/genes15060693 (PMC11202548; doi:10.3390/genes15060693)
Supplement: Supplementary file 1 [file genes-15-00693-s001.zip › TableS1.pdf]

Table S1. Primers used in this experiment

Primer name	Primer sequences (5′-3′)
IbMYB330-F	ATGGGACGTTCTCCTTGCT
IbMYB330-R	CTAAGGAAACCATTTGTAAGAGTTAT
*IbMYB330*pro-F	GCCTTTGTTGGTGTGAGCTT
*IbMYB330*pro-R	CTTGCAGCTATCAACGACCA
RT-IbMYB330-F1	AGAAGAAGACCAACGCCTCA
RT-IbMYB330-R1	CTTGCAGCTATCAACGACCA
IbMYB330-F1	GCGCGGTACCATGGGACGTTCTCCTTGCT
IbMYB330-R1	GCGCGGATCCCTAAGGAAACCATTTGTAAGAGTTAT
IbActin-F	AGCAGCATGAAGATTAAGGTTGTAGCAC
IbActin-R	TGGAAAATTAGAAGCACTTCCTGTGAAC
35S-F	TTGATGTGATATCTCCACTGACG
1300-R	GCGCGGATCCCTAAGGAAACCATTTGTAAGAGTTAT
NtActin-F	GAGGAATGCAGATCTTCGTG
NtActin-R	TCCTTGTCCTGGATCTTAGC
SOD-F	CTATTACCGACAAGCAGATTCCTC
SOD-R	TACCACAAGCAACCCTTCCAC
POD-F	TCCGGGAGCCACACCATTGG
POD-R	TGGTCGGAATTCAACAG
APX-F	GATGTTCCCTTTCACCCTGG
APX-R	CAGATAGACCCATTTGCTTCACA
P5CS-F	TTGTGACACGGACTGATGGAA
P5CS-R	TATCTAAGCCGCTGACGACCA
