# Supplementary figures and images for: A Sweet Potato MYB Transcription Factor IbMYB330 Enhances Tolerance to Drought and Salt Stress in Transgenic Tobacco
Source: Genes (Basel). 2024 May 26;15(6):693. doi: 10.3390/genes15060693 (PMC11202548; doi:10.3390/genes15060693)

(a)

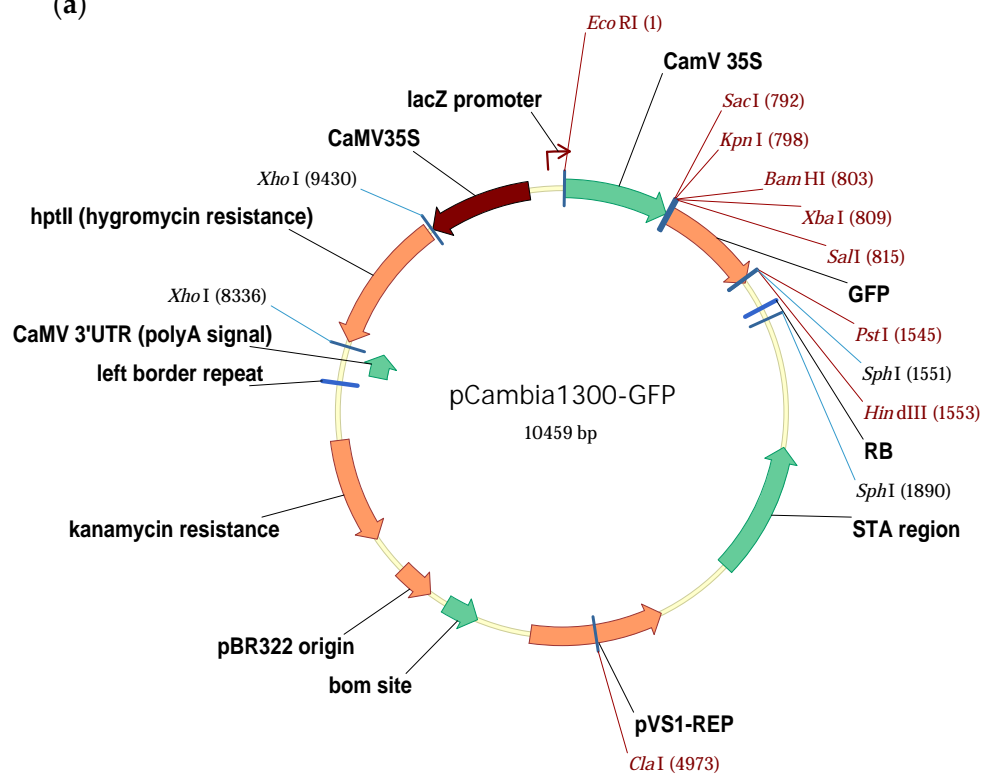

(b)

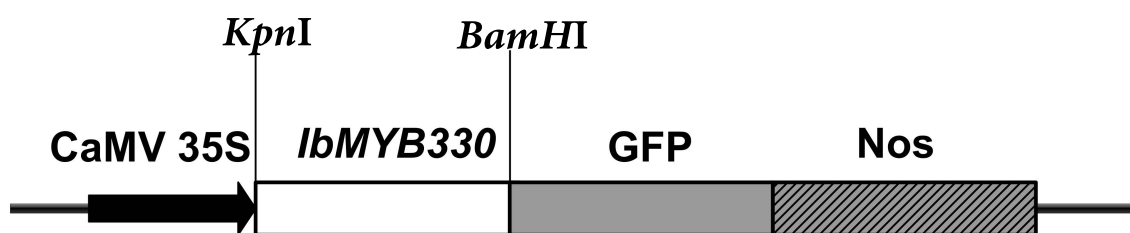

**Figure S1.** (a) pCAMBIA1300 vector, (b) Recombinant plasmid pCAMBIA-*IbMYB330*-GFP

Supplement: Supplementary file 1 [file genes-15-00693-s001.zip › FigureS1.pdf]
